# (-)-Epigallocatechin-3-Gallate Suppresses Hyperexcitability in Rat Primary Nociceptive Neurons Innervating Inflamed Tissues: A Comparison with Lidocaine

**DOI:** 10.3390/metabo15070439

**Published:** 2025-07-01

**Authors:** Syogo Utugi, Yukito Sashide, Mamoru Takeda

**Affiliations:** Laboratory of Food and Physiological Sciences, Department of Life and Food Sciences, School of Life and Environmental Sciences, Azabu University, 1-17-71, Fuchinobe, Chuo-ku, Sagamihara 252-5201, Japan; f22015@azabu-u.ac.jp (S.U.); sashide0112@hotmail.co.jp (Y.S.)

**Keywords:** alternative medicine, extracellular single-unit recording, lidocaine, (-)-epigallocatechin-3-gallate, trigeminal pain, inflammation

## Abstract

**Objective:** Given the side effects and reduced efficacy of conventional local anesthetics in inflammatory conditions, there is a compelling need for complementary alternative medicine (CAM), particularly those based on phytochemicals. While a previous study showed that in vivo local injection of (-)-epigallocatechin-3-gallate (EGCG) into the peripheral receptive field suppresses the excitability of rat trigeminal ganglion (TG) neurons in the absence of inflammation, the acute effects of EGCG in vivo, especially on TG neurons under inflammatory conditions, are still unknown. We aimed to determine if acute local EGCG administration into inflamed tissue effectively attenuates the excitability of nociceptive TG neurons evoked by mechanical stimulation. **Methods:** The escape reflex threshold was measured to assess hyperalgesia caused by complete Freund’s adjuvant (CFA)-induced inflammation. To assess neuronal activity, extracellular single-unit recordings were performed on TG neurons in anesthetized CFA-inflamed rats in response to orofacial mechanical stimulation. **Results:** The mechanical escape threshold was significantly lower in CFA-inflamed rats compared to before CFA injection. EGCG (1–10 mM) reversibly and dose-dependently inhibited the mean firing frequency of TG neurons evoked by both non-noxious and noxious mechanical stimuli (*p* < 0.05). For comparison, 1% lidocaine (37 mM), a local anesthetic, also caused reversible inhibition of the mean firing frequency in inflamed TG neurons responding to mechanical stimuli. Importantly, 10 mM EGCG produced a significantly greater magnitude of inhibition on TG neuronal discharge frequency than 1% lidocaine (noxious, lidocaine vs. EGCG, 19.7 ± 3.3% vs. 42.3 ± 3.4%, *p* < 0.05). **Conclusions:** Local injection of EGCG into inflamed tissue effectively suppresses the excitability of nociceptive primary sensory TG neurons, as indicated by these findings. Significantly, locally administered EGCG exerted a more potent local analgesic action compared to conventional voltage-gated sodium channel blockers. This heightened efficacy originates from EGCG’s ability to inhibit both generator potentials and action potentials directly at nociceptive primary nerve terminals. As a result, EGCG stands out as a compelling candidate for novel analgesic development, holding particular relevance for CAM strategies.

## 1. Introduction

Complementary and alternative medicine (CAM) constitutes a diverse range of therapies distinct from conventional Western medical approaches. The recent literature highlights a growing inclination among patients to utilize CAM modalities, such as herbal medicines and acupuncture, for the alleviation of chronic clinical pain [1,2,3], especially in instances where established medical interventions yield limited efficacy [1,2,3]. Moreover, dietary factors and supplemental intake are recognized as potentially influencing conditions germane to pain [4,5].

The trigeminal nervous system exhibits distinct structures and functions vital for the processing of both orofacial nociception and non-noxious sensations. Orofacial nociception is subserved by small-diameter myelinated Aδ-fibers and unmyelinated C-fibers, which innervate the facial integument, including whisker pads [6]. Nociceptive sensory information originating from the trigeminally innervated territory is conveyed from trigeminal afferents to second-order neurons situated within the brainstem’s spinal trigeminal nucleus (SpV) [6,7]. The SpV, serving as a critical relay station in the transmission of orofacial sensory information, is anatomically and functionally delineated into three subnuclei: oralis, interpolaris, and caudalis [6,7]. Notably, the SpV caudalis (SpVc) functions as a key relay for trigeminal nociceptive inputs generated by inflammation and tissue injury [6,7]. Furthermore, wide dynamic range (WDR) neurons, found in both primary and secondary neuronal orders, are characterized by their responsiveness to both noxious and non-noxious stimulation [8,9,10]. The application of graded nociceptive stimulation to the most sensitive region of their receptive field elicits an increase in firing frequency directly proportional to stimulus intensity. This observation strongly suggests that WDR neurons contribute to the encoding of stimulus intensity within the trigeminal pain pathway.

Green tea is rich in catechins, naturally occurring polyphenols classified as dietary flavonoids. Among these, (-)-Epigallocatechin-3-gallate (EGCG) stands out as a primary and exceptionally active component, accounting for more than 10% of the green tea extract’s dry weight and 65% of its total catechin content [11]. EGCG is recognized for its broad spectrum of beneficial biological activities, such as its antioxidant, anticarcinogenic, and anti-inflammatory attributes [11,12,13,14,15,16]. EGCG influences neuronal excitability by altering various ion channels, including voltage-gated Na (Nav), K(Kv), and Ca(Cav) channels, acid-sensing ion channels (ASICs), and non-selective ion channels. This influence can manifest through mechanisms such as the inhibition of glutaminergic synaptic transmission [17,18,19,20,21,22,23,24,25,26]. Supporting this, Kim et al. [23]) noted that under in vitro whole-cell voltage-clamp conditions, EGCG dose-dependently inhibits both tetrodotoxin-resistant (TTX-R) and -sensitive (TTX-S) Nav channels in the dorsal root ganglion (DRG). Our recent findings demonstrate the effective suppression of excitability in trigeminal ganglion (TG) nociceptive primary sensory neurons following local in vivo injection of EGCG into the peripheral receptive field of rats. This effect is likely mediated by the inhibition of Nav channels and the opening of Kv channels in the nociceptive nerve terminals [27]. Given these results, EGCG, a phytochemical, holds promise as a local anesthetic for trigeminal nociceptive pain in non-neuropathic and non-inflamed conditions. Such an application could offer pain relief with minimal side effects, thus supporting its role in CAM.

Prior research indicates that ASICs could serve as mammalian cutaneous mechanoreceptors in the nerve terminals of trigeminal ganglion (TG) neurons, potentially acting with transient receptor potential ankyrin 1 [28,29,30,31]. Consistent with this, EGCG has been found to inhibit ASIC currents [25]. It is also known that inflamed tissues exhibit a pH below 6.0, a condition that activates primary nociceptive afferents through ASICs [32]. Clinically, inflammation compromises the efficacy of local anesthetics, particularly in dental settings [33]. Supporting this, Fu et al. [34] reported that peripheral inflammation upregulates ASICs in TG neurons and that specific ASIC inhibitors exert significant analgesic effects on orofacial inflammatory pain. Taken together, these findings strongly suggest that local administration of EGCG directly inhibits generator potentials and consequently suppresses the action potential firing of nociceptive TG neurons in inflamed tissues. This inhibition is likely mediated via the concurrent inhibition of ASIC channels, Nav channels, and the opening of Kv channels. Consequently, EGCG may offer relief from trigeminal inflammatory pain with minimal side effects, thereby contributing to CAM. However, the acute in vivo effects of EGCG under inflammatory conditions, particularly on nociceptive transmission within the trigeminal system, warrant further investigation for full determination. Therefore, the present study investigated whether acute local administration of EGCG into inflamed tissues attenuates the excitability of nociceptive TG neurons in response to mechanical stimulation during inflammation. Furthermore, we compared EGCG’s potency in suppressing trigeminal inflammatory pain with that of lidocaine, a conventional, clinically-used local anesthetic and Na channel blocker, to evaluate EGCG’s potential contribution to CAM. Local administration of the phytochemical EGCG into inflamed tissues was found to elicit a more potent local analgesic effect than voltage-gated sodium channel blockers. This superior efficacy stems from EGCG’s ability to inhibit both generator potentials—possibly through ASIC blockade—and action potentials in nociceptive primary nerve terminals. Consequently, EGCG holds significant promise for contributing to CAM.

## 2. Materials and Methods

All experiments in this report were approved by the Animal Use and Care Committee of Azabu University (No. 230120-12) and strictly adhered to the ethical guidelines of the International Association for the Study of Pain [35]. We minimized both the number of animals used and their suffering.

### 2.1. Preparing Animals and Inducing Inflammation

Adult male Wistar rats (205–235 g) were acclimatized to a controlled environment, including a fixed 12 h light/dark cycle (lights on 07:00–19:00) and a room temperature maintained at 23 ± 1 °C. They had ad libitum access to food and water. For electrophysiological recording, 15 rats were utilized. Each animal underwent anesthesia with 3–5% isoflurane, after which 0.05 mL of a 1:1 oil/saline suspension of complete Freund’s adjuvant (CFA) was unilaterally injected into the left facial skin, consistent with a previously established protocol [36]. Behavioral assessments were performed at two time points: prior to the CFA injection and one day post-injection.

### 2.2. Measurement of Mechanical Escape Threshold and Inflammatory Edema

**Figure 1:**
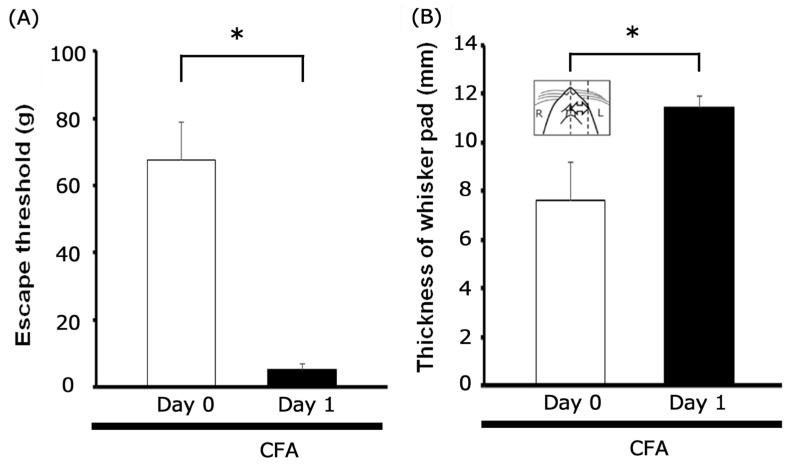
Post-inflammation changes in mechanical threshold and inflammatory edema. (**A**) Mechanical hyperalgesia was assessed by applying von Frey filaments to the ipsilateral whisker pad of rats on Day 1 following complete Freund’s adjuvant (CFA) injection. Data are presented as mean ± SEM; * *p* < 0.05 when comparing inflamed Day 0 vs. inflamed Day 1 (*n* = 15). (**B**) Mean thickness of the edematous whisker pad area before and after ipsilateral CFA injection. * *p* < 0.05 (*n* = 15). Inset: area designated for measuring CFA-induced orofacial inflammatory edema.

Mechanical withdrawal thresholds were assessed as previously described [36]. Briefly, mechanical hyperalgesia was evaluated by applying a set of von Frey hairs (Semmes-Weinstein Monofilaments; North Coast Medical, Morgan Hill, CA, USA) to the ipsilateral and contralateral facial skin regions starting one day after CFA injection. Escape thresholds in rats were established using an ascending series of von Frey mechanical stimuli (0.06–100 g) applied to the whisker pad, with each stimulus administered thrice per series. The threshold intensity was defined as the minimum force eliciting a head withdrawal response to at least one of the three stimuli. For evaluating EGCG’s impact on peripheral inflammation, CFA injection-induced edema in the whisker pad was quantified. This quantification involved measuring the thickness of the edematous region pre- and post-CFA injection (Figure 1B, inset), consistent with prior descriptions [36].

### 2.3. Extracellular Single-Unit Electrophysiology of TG Neurons

Each rat received an initial anesthetic induction with 3–5% isoflurane, followed by a combination anesthetic consisting of 0.3 mg/kg medetomidine, 4.0 mg/kg midazolam, and 5.0 mg/kg butorphanol. Anesthesia was subsequently maintained via a jugular vein cannula, with supplemental doses administered as required to ensure adequate depth throughout the recording session, confirmed by the absence of a paw pinch reflex. Body temperature was precisely controlled at 37.0 ± 0.5 °C using a homeothermic blanket (Temperature Controller, 40-90-8D; FHC, Aspen, Tokyo, Japan). To minimize discomfort, all wound margins were continuously perfused with 2% lidocaine (Xylocaine) during the entire experimental procedure. Following anesthesia, animals were positioned in a stereotaxic apparatus (SR-50; Narishige, Tokyo, Japan) for a partial craniotomy, performed at coordinates 2–5 mm posterior to bregma and 1–4 mm lateral to the midline. An enamel-coated tungsten microelectrode (impedance = 3–5 MΩ) was meticulously positioned through the cortex at coordinates approximately 2.5–3.5 mm lateral to the midline and 2.5–3.5 mm posterior to bregma (depth 8.1–9.9 mm), aiming to reach the TG, as detailed in previous publications [27,36,37]. Precise isolation of single-unit activity was achieved by advancing or retracting the electrode in 10 μm increments using a micromanipulator (SM-11 and MO-10; Narishige). Extracellular single-unit activity from the TG region was captured utilizing the tungsten electrode, with placement guided by the stereotaxic coordinates derived from Paxinos and Watson [38]. The acquired neuronal activity signals were subsequently amplified (DAM80; World Precision Instruments, Sarasota, FL, USA), band-pass filtered (0.3–10 kHz), continuously monitored on an oscilloscope (Iwatsu, SS-7672, Tokyo, Japan), and digitally recorded for comprehensive off-line analysis via PowerLab and Chart v.5 software (ADI Instruments, Oxford, UK), in accordance with established methods [27,36,37].

### 2.4. Experimental Protocol: Electrophysiological Recording

Extracellular single-unit activity of TG neurons was recorded following mechanical stimulation of the whisker pad. To mitigate the sensitization of peripheral mechanoreceptors, an initial coarse mapping of the receptive field on the left whisker pad was conducted using a rapid paintbrush stimulus [27,36,37]. Subsequently, individual units on the left whisker pad were thoroughly screened for responses to a calibrated series of von Frey hairs, with non-noxious (1, 2, 4, 6, 8, and 10 g) and noxious (15, 26, and 60 g) mechanical stimuli applied for 5 s at 5 s inter-stimulus intervals [27,36,37]. WDR neurons were characterized by their graded increase in firing frequency in direct proportion to the intensity of both non-noxious and noxious mechanical stimulation applied to their receptive field. Upon identification of nociceptive TG WDR neurons responsive to whisker pad stimulation, their mechanical stimulation threshold and receptive field dimensions were subsequently determined. The mechanical receptive field of neurons was mapped by probing the facial skin with von Frey hairs; the field’s boundaries were then outlined on a life-sized drawing of the rat using tracing paper [27,36,37]. TG neuronal discharges in response to mechanical stimulation were quantified by subtracting background activity from the evoked activity. Spontaneous discharge frequencies were determined from recordings over 2–5 min. Post-stimulus histograms (100 ms bin width) were generated for each stimulus. We evaluated the effects of subcutaneously administered EGCG and lidocaine. EGCG (Sigma-Aldrich, Milano, Italy; 1 mM, 0.02 mL) and lidocaine (1% injection solution, equivalent to 37 mM, 0.02 mL; lidocaine HCl, 2-Diethylamino-N-[2,6-dimethylphenyl]acetamide, MW = 280.1, pH 5.0–7.0; Aspen Japan) were delivered via a Hamilton micro syringe. Effects were assessed at 5, 10, 15, 20, 30, 40, 45, 50 and 60min post-administration, as this timeframe was expected to encompass peak effect and recovery. EGCG stock solutions (1 mM and 10 mM) were prepared by dissolving EGCG in 100% dimethyl sulfoxide (DMSO) and stored at −20 °C until use. In this study, we analyzed the mean spontaneous and mechanical stimulation-induced discharge rates, as well as the mechanical threshold, before and after the subcutaneous administration of EGCG and lidocaine.

### 2.5. Data Analysis and Statistics

Data are presented as the mean ± standard error of the mean (SEM). Statistical analyses involved one-way repeated-measures analysis of variance (ANOVA). Post hoc analyses were subsequently conducted using Tukey–Kramer or Dunnett’s tests. Additionally, *t*-tests were utilized for certain comparisons. These analyses were applied to both behavioral and electrophysiological data (Excel Statcel 4). A two-sided *p*-value of <0.05 was considered to indicate statistical significance.

## 3. Results

### 3.1. Characterization of Inflammation-Induced Hyperalgesia and Edema

Our initial assessment involved testing for hyperalgesia in inflamed rats by applying von Frey filaments to the CFA-injected site and/or the orofacial skin (whisker pad). We observed that CFA administration significantly lowered the mechanical withdrawal threshold in the whisker pad area from 67.7 ± 11.2 g in non-injected animals to 5.3 ± 1.6 g on Day 1 post-injection (*n* = 15, *p* < 0.05; Figure 1A). Importantly, no significant alterations were detected in the contralateral whisker pad threshold when comparing pre- and post-injection measurements (65.2 ± 8.7 g vs. 63.1 ± 6.6 g, *n* = 15, not significant). Concurrently, the mean thickness of the edematous region within the whisker pad of CFA-inflamed rats was significantly elevated relative to pre-CFA injection values (7.6 ± 1.6 mm vs. 11.4 ± 0.5 mm, *n* = 15, *p* < 0.05; Figure 1B).

### 3.2. Physiological Properties of Trigeminal Ganglion Neurons Innervating Inflamed

#### Facial Skin

**Figure 2:**
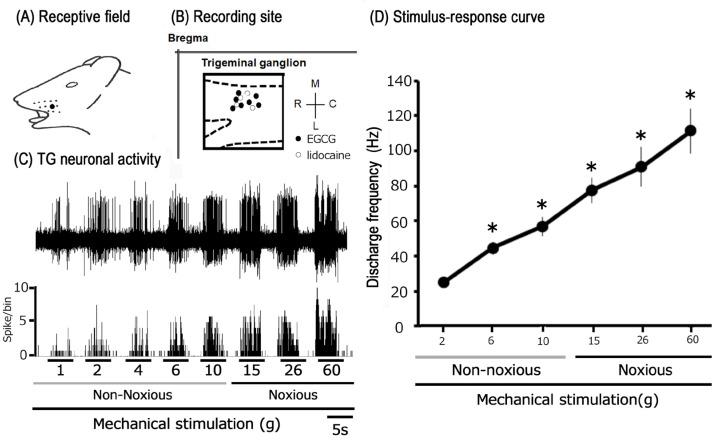
Characterization of trigeminal ganglion (TG) neuronal activity and the impact of peripheral subcutaneous EGCG/lidocaine on responses to mechanical stimulation in inflamed states. (**A**) Representative receptive field located on the whisker pad area of the facial skin (blackened). (**B**) Positional distribution of TG neurons (*n* = 15) that responded to non-noxious and noxious mechanical stimulation applied to the inflamed facial skin. The inset provides an example of how TG recording sites were identified (2–5 mm posterior to bregma and 1–4 mm lateral to midline). R: rostral; C: caudal; M: medial; L: lateral. Trigeminal nerve (I, II, and III). (**C**). Representative examples of TG neuronal responses to non-noxious (2, 6, 10 g) and noxious (15, 26, 60 g) mechanical stimulation of the orofacial skin. The upper trace shows TG neuronal activity, while the lower trace depicts the post-stimulus histogram. (**D**) Stimulus–response curve of SpVc WDR neurons (*n* = 15). * *p* < 0.05 for comparisons between the 2 g stimulus and 6 g, 10 g, 15 g, 26 g, and 60 g stimuli.

Subsequently, extracellular single-unit recordings were conducted from 15 TG neurons in CFA-inflamed Day 1 rats. The impact of subcutaneous EGCG injections was evaluated on eleven TG neurons (1 mM, *n* = 4; 10 mM, *n* = 7). Furthermore, four neurons were utilized to investigate neuronal excitability following subcutaneous administration of the Nav channel blocker, lidocaine (1%). A representative example of an inflamed receptive field of TG neurons in the whisker pad, exhibiting responses to both non-noxious and noxious mechanical stimuli, is presented in Figure 2A. The recording locations for single units were predominantly situated within the maxillary branches of the TG (Figure 2B) [27,36,37]. The recording site locations did not differ significantly between the EGCG- and lidocaine-injected groups (Figure 2B). Figure 2C illustrates representative examples of TG neuronal unit responses. Applying graded mechanical stimulation to the most sensitive region of the receptive field elicited an increase in the firing frequency of TG WDR neurons that was directly proportional to the stimulus intensity. Consistent with our prior investigations [27,36,37], ten of the fifteen TG neurons (71.4%) exhibited spontaneous discharges (5.5 ± 2.2 Hz). The mean spike threshold induced by mechanical stimulation was 1.3 ± 0.7 g. Notably, every neuron recorded was classified as a WDR neuron [27,36,37].

### 3.3. Modulation of Trigeminal Ganglion Neuronal Responses to Mechanical Stimuli by Lidocaine Following CFA-Induced Inflammation

**Figure 3:**
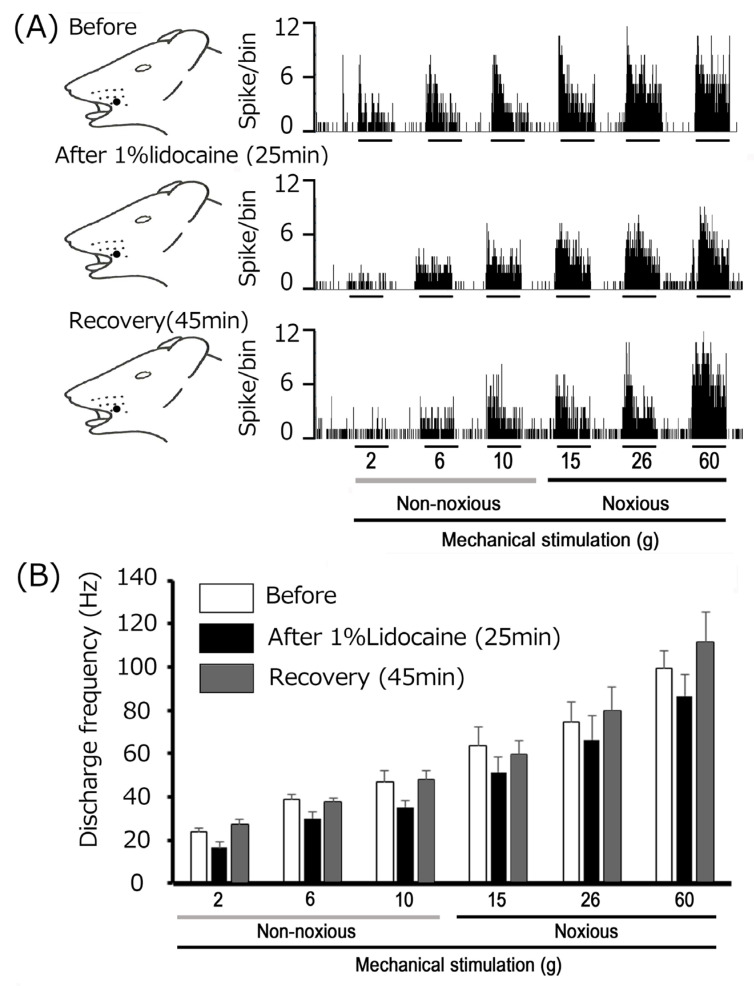
Modulation of trigeminal ganglion (TG) neuronal responses to mechanical stimulation by subcutaneous lidocaine (1%, 37 mM) in the peripheral receptive field. (**A**) Effects of peripheral subcutaneous lidocaine (1%, 37 mM) administration on trigeminal ganglion (TG) neuronal responses to mechanical stimulation. Responses were recorded at baseline (before) and at 25 and 45 min post-1% lidocaine administration. The receptive field was situated on the whisker pad of the facial skin, and the blackened region indicates its precise location and extent. (**B**) Time-course of the effect of local lidocaine administration into the peripheral receptive field on the mean firing frequency of inflamed TG neurons responsive to non-noxious and noxious mechanical stimulation (*n* = 4). There was no significant difference in firing frequency when comparing before and 25 min post-1% lidocaine administration, nor when comparing 25 min and 45 min post-administration.

To compare the potency of EGCG with a conventional, clinically used local anesthetic in suppressing trigeminal inflammatory pain, the effect of lidocaine injection on the excitability of inflamed TG neurons was first investigated. Figure 3 presents representative examples of the effects of 1% lidocaine (37 mM), subcutaneously injected into the center of the receptive field, on the excitability of inflamed TG neurons responding to non-noxious and noxious mechanical stimulation. The response of inflamed TG neurons to both non-noxious and noxious mechanical stimulation was inhibited for approximately 25 min after lidocaine injection, with responses returning to control levels within 45 min (Figure 3), consistent with our previous study [36]. Figure 3 illustrates the effects of lidocaine injection on mechanical stimulation-evoked TG neuronal activity in inflamed rats. Following EGCG injection, the mean firing rates of TG neurons evoked by non-noxious and noxious mechanical stimulation tended to decrease compared with controls and also returned to control levels within 45 min (Figure 3).

### 3.4. Modulation of Trigeminal Ganglion Neuronal Responses to Mechanical Stimuli by EGCG Following CFA-Induced Inflammation

**Figure 4:**
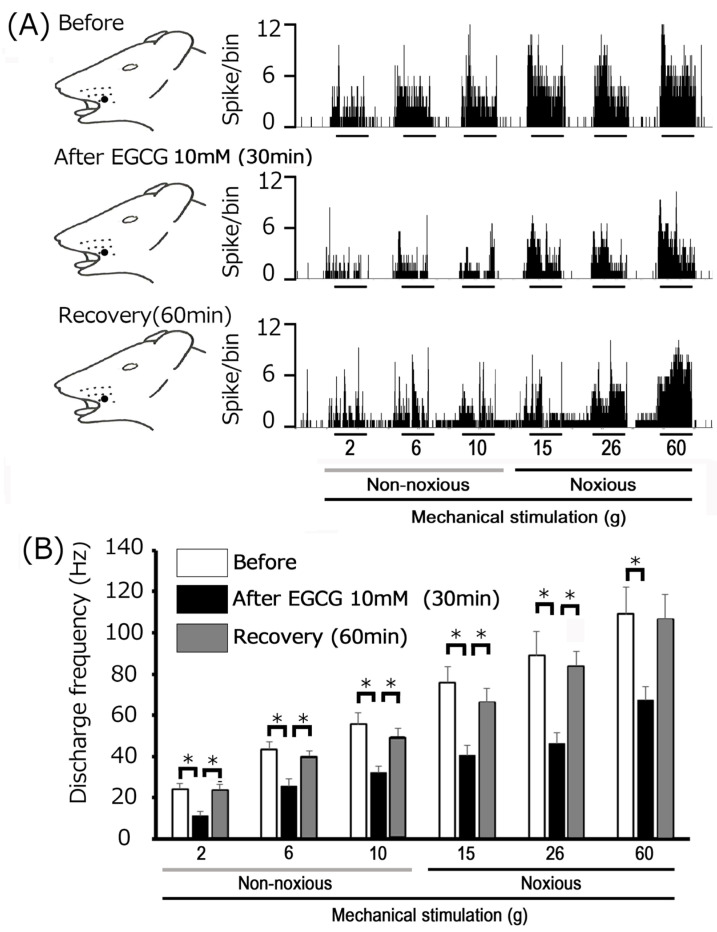
Influence of subcutaneous EGCG administration into the peripheral receptive field on trigeminal ganglion neuronal responses to noxious and non-noxious mechanical stimuli in CFA-inflamed rats. (**A**) Representative examples of TG WDR neuronal activity elicited by non-noxious (2, 4, and 10 g) and noxious (15, 26, and 60 g) mechanical stimuli. Responses were recorded at baseline (before) and at 30 and 60 min post-10 mM EGCG administration. (**B**) Time-course of the impact of local EGCG administration into the peripheral receptive field on the mean firing frequency of TG neurons responsive to non-noxious and noxious mechanical stimulation. * *p* < 0.05 when compared to the 30 min post-EGCG administration time point (*n* = 7).

Figure 4A illustrates representative examples of the impact of local subcutaneous EGCG injection (10 mM) on the excitability of inflamed TG neurons in response to non-noxious mechanical stimulation. Non-noxious (2–10 g) mechanical stimulation-evoked TG neuronal activity was significantly inhibited 30 min after subcutaneous injection of 10 mM EGCG into the receptive field’s center, with activity recovering to control levels within approximately 60 min. Neither the mechanical threshold nor the receptive field size exhibited discernible changes following EGCG administration. Although a trend toward decreased spontaneous discharge of TG neurons was observed after EGCG injection, this effect did not reach statistical significance. Figure 4B illustrates how EGCG affects TG neuronal activity evoked by non-noxious mechanical stimulation. EGCG injection significantly reduced the mean firing rates of TG neurons (2, 6, and 10 g) compared to pre-injection levels (*p* < 0.05, *n* = 7). Notably, these attenuated discharges significantly recovered to control levels within 45 min (*n* = 7).

**Figure 5:**
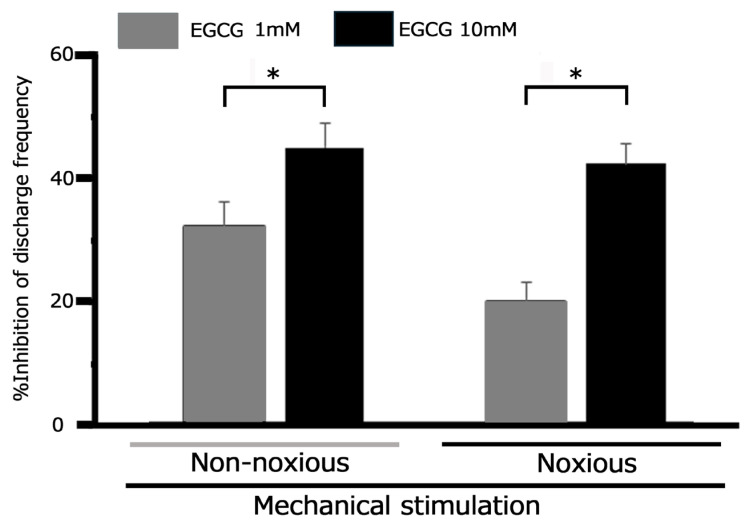
EGCG’s dose-dependent suppression of TG neuronal firing frequency under inflamed conditions in response to mechanical stimulation. * *p* < 0.05, 1 mM (*n* = 4) vs. 10 mM (*n* = 7).

Representative examples in Figure 4A show how subcutaneous injection of 10 mM EGCG into the receptive field alters the excitability of inflamed TG neurons during noxious mechanical stimulation. Noxious (15, 26, and 60 g) mechanical stimulation-evoked TG neuronal activity was inhibited 30 min post-EGCG injection, with neuronal activity recovering to control levels within approximately 60 min (Figure 3A). The mean firing rates of TG neurons elicited by noxious (15, 26, and 60 g) mechanical stimulation demonstrated a significant decrease after EGCG injection compared to control conditions (*p* < 0.05; *n* = 7; Figure 3B), and neuronal activity returned to control levels within approximately 45 min (60 g, *p* < 0.05; *n* = 7). Consistent with our previous findings [36], local injection of vehicle (dimethyl sulfoxide) had no significant effect on trigeminal ganglion (TG) neuronal activity evoked by either non-noxious or noxious mechanical stimulation. Specifically, for non-noxious stimulation (6 g), mean firing rates were 43.1 ± 4.2 Hz before vs. 45.1 ± 6.1 Hz after injection. For noxious stimulation (60 g), rates were 111.2 ± 9.8 Hz before vs. 108.5 ± 14.3 Hz after injection. EGCG’s inhibitory effect on non-noxious stimulation was dose-dependent (1 mM, *n* = 3, vs. 10 mM, *n* = 7, *p* < 0.05; Figure 5). Similarly, EGCG inhibited noxious stimulation in a dose-dependent manner (1 mM, *n* = 3, vs. 10 mM, *n* = 7, *p* < 0.05, Figure 5). Notably, the maximum peak of inhibition for TG neuronal discharge frequency after 10 mM EGCG injection occurred more rapidly (within 10 min) than with 1 mM EGCG.

### 3.5. Effect of EGCG on Trigeminal Ganglion Neuronal Activity Following CFA-Induced Inflammation: Response to Noxious and Non-Noxious Stimuli

To assess the inhibitory potency of a subcutaneous 10 mM EGCG injection in inflamed tissue, we compared its effect on responses to non-noxious and noxious stimuli. EGCG produced a similar mean magnitude of inhibition for both non-noxious and noxious stimulation-induced discharge frequency (non-noxious vs. noxious: 44.8 ± 3.3% vs. 42.3 ± 3.4%, NS, *n* = 7). In contrast, the percentage inhibition frequency of noxious stimulation-induced discharge by 10 mM EGCG differed significantly between naïve and CFA-inflamed animals. (naïve vs. inflamed: 58.0 ± 3.0% vs. 42.3 ± 3.4%, *p* < 0.05), with reference to our prior findings [27].

### 3.6. Comparative Analysis of EGCG and Lidocaine on TG Neuronal Responses to Mechanical Stimuli Following CFA-Induced Inflammation

**Figure 6:**
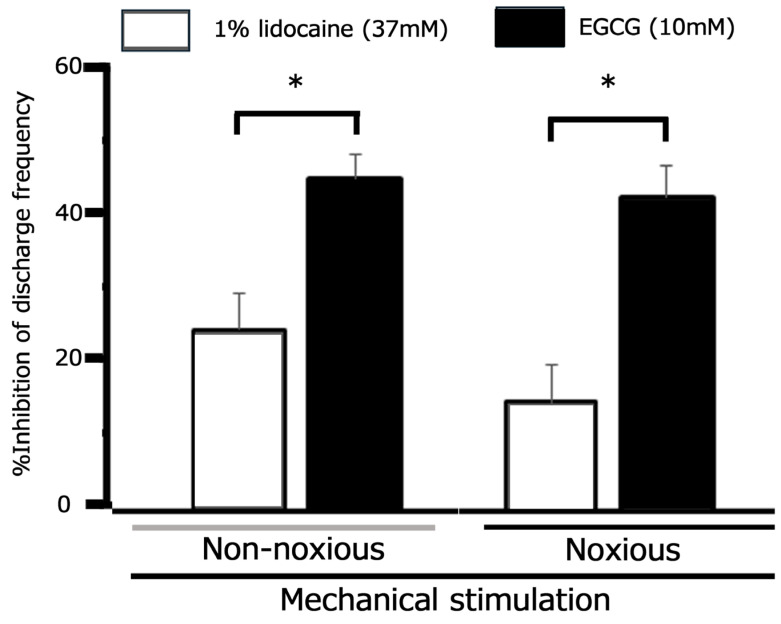
Comparative analysis of the relative inhibitory efficacy of EGCG and lidocaine on trigeminal ganglion neuronal discharge. * *p* < 0.05, 1% lidocaine (*n* = 4) vs. 10 mM EGCG (*n* = 7).

Ultimately, we aimed to compare the inhibitory efficacy of 10 mM EGCG and 1% lidocaine (37 mM) on TG neuronal discharge frequency. Our findings, summarized in Figure 6, show that EGCG exhibited a significantly greater mean magnitude of inhibition on inflamed TG neuronal discharge evoked by non-nociceptive stimulation than 1% lidocaine. This superior inhibitory effect of EGCG was also observed for inflamed TG neuronal discharge elicited by nociceptive stimulation (Figure 6).

## 4. Discussion

### 4.1. Local EGCG Application Suppresses Hyperexcitability in Nociceptive Primary Trigeminal Ganglion (TG) Neurons Innervating Inflamed Tissues

The impairment of local anesthetic efficacy by inflammation is a widely recognized phenomenon, particularly relevant to dental anesthesia [33]. Previously, we demonstrated that local EGCG administration into the peripheral receptive field effectively modulates the excitability of nociceptive primary sensory neurons in the TG [27]. However, the efficacy of EGCG in modulating nociceptive primary neuronal activity under inflammatory conditions had not yet been characterized. Thus, the present study aimed to ascertain whether acute local administration of EGCG could attenuate the excitability of nociceptive TG neurons evoked by non-noxious and noxious mechanical stimulation under inflammatory conditions. This investigation yielded the following principal findings: (i) Day 1 CFA-inflamed rats exhibited a significantly reduced mean mechanical escape threshold relative to their pre-CFA baseline, demonstrating mechanical hyperalgesia. (ii) The mean thickness of the edematous area in the whisker pad significantly increased in CFA-inflamed rats compared to pre-CFA injection measurements. (iii) As observed in our previous studies, CFA-inflamed rats exhibited reaffirmed hyperexcitability, marked by a decreased mechanical threshold and increased spontaneous and mechanically induced discharges. (iv) In Day 1 inflamed rats, EGCG significantly and dose-dependently attenuated the mean firing frequency of TG neurons in response to both non-noxious and noxious mechanical stimuli. This maximal inhibition of discharge frequency occurred within 45 min, persisted for 60 min, and proved to be reversible for both types of stimuli. (v) Vehicle administration exerted no significant effect on the discharge frequency elicited by non-noxious or noxious mechanical stimuli. In the present experiment, we substantiated EGCG’s inhibitory effect on TG neuronal discharge frequency in response to noxious stimulation in both inflamed and normal animals, consistent with our prior publications [27]. These findings collectively suggest that localized EGCG administration into the peripheral receptive field of inflamed tissue can effectively mitigate the excitability of primary sensory neurons. This outcome is further corroborated by our previous investigation, which demonstrated that local application of another phytochemical, quercetin, reversibly and dose-dependently inhibits TG neuronal activity in inflamed tissues [36]. Additional confirmatory studies remain essential, however. These include in vitro patch-clamp investigations employing dissociated, fluorescently-labeled TG neurons isolated from the receptive field of inflammatory tissues [39].

### 4.2. Peripheral Mechanism: How Local EGCG Administration Suppresses TG Neuronal Excitability in Inflamed Tissues

The pathways of nociceptive sensory signaling proceed through several sequential steps, beginning with signal transduction from peripheral terminals that convert external stimuli, followed by the generation and propagation of action potentials along axons culminating in their transmission to central terminals, which constitute the presynaptic components of the initial synapses in the central nervous system sensory pathways [7,40]. Peripheral sensitization describes the phenomenon where the peripheral terminals of high-threshold primary sensory neurons experience a reduced threshold and increased responsiveness upon exposure to inflammatory mediators and tissue damage [41]. After inflammation and nerve injury, inflammatory mediators such as prostaglandin E2 bind to G-protein-coupled receptors. This binding induces the activation of protein kinase A and C within the nociceptors of peripheral terminals, leading to the phosphorylation of ASIC, Nav, and Kv channels, as well as various receptors [34,42]. As a result, alterations in the activation threshold for transducer channels, like ASICs, and analogous changes in Nav and Kv channel functions are significant contributors to inflammatory pain. These changes are largely attributed to the hyperexcitability exhibited by nociceptive neurons, marked by a diminished activation threshold and an accelerated action potential firing rate [43,44,45,46,47,48].

The present study demonstrates that local in vivo EGCG injection into the inflamed peripheral receptive field effectively suppresses the excitability of nociceptive primary sensory TG neurons. Given EGCG’s established inhibition of ASIC currents in cultured neurons [25], it is reasonable to hypothesize that locally administered EGCG operates through ASICs, which are considered candidate mechanoreceptors in the nerve terminals of TG neurons [49,50]. Supporting this, a recent report demonstrated that peripheral inflammation leads to ASIC upregulation in TG neurons, and specific ASIC inhibitors can significantly alleviate orofacial inflammatory pain [34]. However, our study did not investigate whether pretreatment with a specific ASIC antagonist attenuates the EGCG-induced discharge frequency of TG neurons in response to both non-noxious and noxious mechanical stimulation. Therefore, we were unable to determine if EGCG preferentially inhibits ASIC3, a nociceptive mechanoreceptor, rather than Nav channels. This possibility warrants further investigation. Taken together, our results suggest that local EGCG administration directly inhibits generator potentials under inflamed conditions, thereby suppressing the action potential firing of nociceptive TG neurons.

EGCG has been reported to modulate Nav, Kv, and Cav channels in excitable tissues, influencing cell excitability. [21,22,26]. Black et al. [51] demonstrated that chronic inflammation leads to the upregulation of both TTX-S and TTX-R Nav channels. While TTX-R Nav channels are selectively expressed in nociceptive dorsal root ganglion (DRG) neurons (analogous to Aδ/C-primary afferent TG neurons), TTX-S Nav channels are present in Aβ/Aδ-primary afferent TG neurons [21,52,53]. Intriguingly, Wallace et al. [21], a study employing a whole-cell patch clamp technique in rat cardiac myocytes, reported that the red grape polyphenols quercetin, catechin, and resveratrol inhibit Na currents, exhibiting a half-maximal inhibitory concentration order of quercetin > catechin > resveratrol. Similarly, in the current study, we observed that 10 mM EGCG significantly inhibited the mean firing frequency of TG neurons evoked by both non-noxious and noxious mechanical stimuli in Day 1 inflamed rats. Importantly, the suppression of nociceptive-evoked TG neuron firing achieved with 10 mM EGCG in this study was less pronounced than that previously observed with 10 mM quercetin in our laboratory (EGCG vs. quercetin; 42% vs. 72%) [36]. This outcome substantiates the findings reported by Wallace et al. [21]. Collectively, these observations suggest that EGCG injected into inflamed tissue suppressed the upregulated currents of both TTX-S and TTX-R Nav channels.

Consistent with this, our prior investigations revealed that under inflammatory conditions, a diminished current density of slow-inactivating sustained (K-current) and fast-activating transient (A-current) channels contributes to the augmented excitability of small-diameter TG neurons in CFA-inflamed rats [53,54]. We further reported that temporomandibular joint (TMJ) inflammation reduces voltage-gated K^+^ channel subtype Kv1.4-immunoreactivity in Aδ/C-TG neurons in rats [54]. Considering that in vivo application of an A-type Kv channel blocker to TG neurons similarly enhances Aδ/C-TG neuronal activity innervating the TMJ region in intact rats [55], the A-type Kv channel in Aδ/C-TG neurons innervating the TMJ is pivotal in trigeminal inflammatory pain observed in TMJ disorders. The administration of EGCG has been shown to induce vasorelaxation via the potentiation of Kv channels and suppression of Cav channels in smooth muscle [22,26]. Moreover, Granados-Soto et al. [56] demonstrated in an in vivo rat formalin test that dietary constituents, such as resveratrol, elicit peripheral antinociception through the activation of various Kv channels. Given that the opening of Kv channels results in the hyperpolarization of resting membrane potentials and, consequently, a reduction in cellular excitability, several Kv channel subtypes have been posited as promising therapeutic targets for pain [53,54]. These findings suggest that local EGCG injection into the inflamed peripheral receptive field attenuates the excitability of TG neurons responsive to noxious mechanical stimulation, possibly by activating Kv channels in their nociceptive nerve terminals. Nevertheless, this hypothesis requires substantiation through additional in vitro investigations.

T-type Cav channels in primary sensory afferents of the pain pathway sustain neuronal firing and may contribute to neurotransmitter release at spinal dorsal horn afferent terminals, as indicated by previous studies [57,58]. This heightened neuronal excitability leads to an amplification of sensory transmission, ultimately resulting in pathological pain perception [59]. Consequently, blocking T-type Cav/Cav3.2 channels has been shown to mediate analgesia [60]. The flavonoid gossypetin, which shares significant structural similarity with quercetin, was shown by Gadotti et al. [60] to inhibit inflammatory and neuropathic peripheral pain states, partly via its action on Cav3.2 channels. Toyota et al. [37] previously demonstrated that in vivo local injection of quercetin into the peripheral receptive field suppresses the excitability of nociceptive primary sensory neurons in the TG, possibly via the inhibition of Nav and T-type Cav channels and the opening of Kv channels in the nociceptive nerve terminals. The work by Gambeta et al. [59,61] revealed that T-type calcium channels critically regulate neuronal activity in the trigeminal system, thereby profoundly influencing trigeminal pain. Ali et al. [62] recently demonstrated that flavonols, such as quercetin, inhibit inflammatory and neuropathic pain through a dual mechanism involving T-type Cav channels: direct inhibition and alteration of deubiquitination. Notably, quercetin was found to be a highly effective disruptor of the USP5 (deubiquitinase)/Cav3.2 interaction. Therefore, these findings collectively suggest that EGCG, a flavonoid with effects analogous to quercetin, may inhibit the excitability of trigeminal neuronal firing induced by noxious mechanical stimulation. This could occur through the blockade of T-type Cav channels under inflammatory conditions. Nevertheless, this possibility warrants further in vitro investigation for complete elucidation.

### 4.3. Local EGCG Administration Suppresses Nociceptive Hyperexcitability in Inflamed TG Neurons: Functional Implications

The use of CAM therapies for pain management is widespread, especially in instances of conventional Western medicine failure [1,2,3] or owing to concerns about potential adverse side effects [4,5]. The use of local anesthetic agents carries a risk of adverse effects on the central nervous system or cardiovascular system if systemic blood levels become too elevated [63]. Consequently, recent research indicates an increased interest in efficacious CAM therapies for managing pain [36,37].

The CFA-induced orofacial pain model in rats is widely utilized to investigate trigeminal pathological pain, such as inflammation-induced mechanical hyperalgesia [53,64]. Prior research indicates that peripheral CFA-induced inflammation can lead to the upregulation of Nav and Cav channels and the downregulation of Kv channels in primary sensory neurons, including TG neurons [7,45]. Therefore, it is crucial to investigate whether local EGCG application attenuates CFA-induced inflammatory hyperexcitability of TG neurons responsive to nociceptive mechanical stimulation. Our current study confirmed mechanical hyperalgesia in CFA-injected Day 1 rats. Clinically, it is well-documented that inflammation reduces local anesthetic efficacy, a phenomenon frequently observed in dentistry [24]. In inflamed tissues, a decrease in pH alters the equilibrium between ionized and non-ionized lidocaine molecules. This relative reduction in non-ionized lidocaine subsequently impairs its binding to Nav channels, thereby weakening the effectiveness of local anesthetics under inflammatory conditions. In line with this, we observed that 1% lidocaine reversibly inhibited the mean firing frequency of inflamed TG neurons in response to both non-noxious and noxious mechanical stimuli in the present study. Crucially, 10 mM EGCG achieved a significantly greater magnitude of inhibition on TG neuronal discharge frequency than 1% lidocaine. Furthermore, the peak inhibition by 10 mM EGCG was sustained for a longer duration than that by 1% lidocaine (30 min vs. 25 min). As noted earlier, EGCG and lidocaine interact with different molecular targets and possess varying affinities. While lidocaine is a selective and potent Nav channel blocker [65], EGCG modulates a broader range of voltage-gated channels (e.g., Nav, Kv, and Cav) and mechanosensitive ion channels, including ASIC3 [21,22,25,26]. Consequently, the comparatively extended duration of inhibition by EGCG compared to lidocaine may stem from differences in their physicochemical properties and the fact that only lidocaine acts as a selective Nav channel blocker.

Our previous work demonstrated that the inhibitory effect of EGCG on mechanical stimulation-induced discharge persisted longer in inflamed rats compared to naive rats (30 min vs. 20 min) [27]. In this study, we confirmed that CFA injection led to a significantly larger mean thickness of the edematous area in the whisker pad of rats. We administered EGCG directly into this edematous swelling. Although the exact mechanism underlying the difference in inhibitory effect duration between naive and inflamed rats is yet to be elucidated, it is plausible that variations in drug infiltration into the injection sites contribute to the observed differences in maximum inhibitory peak times, as suggested by prior research. Crucially, this study revealed that EGCG functions as a potent local anesthetic for inflammatory tissue, demonstrating an advantage over lidocaine. This finding strongly suggests that elucidating the chemical structure of EGCG could be pivotal in designing next-generation anesthetics for inflammatory conditions. Comprehensive experimental and clinical validation is, however, indispensable to fully realize this therapeutic potential.

**Figure 7:**
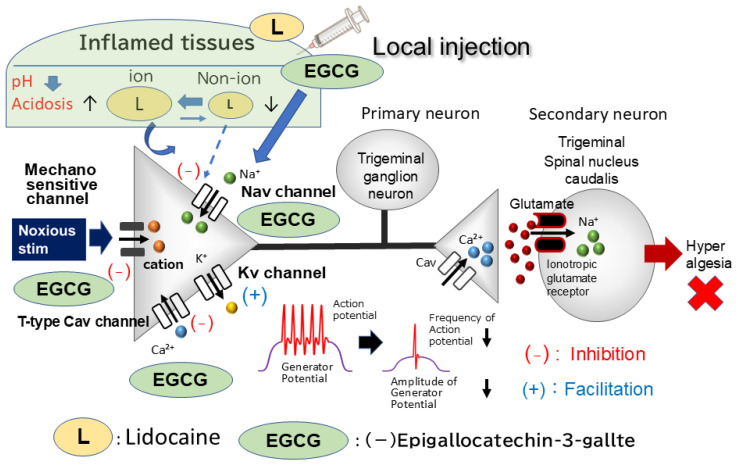
Mechanisms underlying local anesthetic effect of EGCG under inflammatory conditions. Within inflammatory tissues, the local acidic environment, characterized by a lower pH, shifts the equilibrium of lidocaine towards its ionized form. This results in a decreased proportion of the non-ionized species, which is the form primarily responsible for binding to Nav channels. Consequently, the reduced binding leads to a diminished anesthetic effect in inflamed tissues. EGCG’s inhibitory effect on inflamed tissues likely arises from its multi-faceted action, including the suppression of action potential firing frequency through inhibiting nociceptive mechanosensitive channels (ASICs), Nav channels, and T-type Cav channels, in addition to opening Kv channels. EGCG’s inhibitory potency on discharge frequency is significantly higher than lidocaine’s, indicating its strong local anesthetic effect on inflamed tissues. Therefore, EGCG holds promise for use in complementary and alternative medicine. Abbreviations: TG = trigeminal ganglion, Nav = voltage-gated sodium channel, Kv = voltage-gated potassium channel, Cav = voltage-gated calcium channel, SpVc = spinal trigeminal nucleus caudalis, ASIC = acid-sensing ion channel, TRPA1 = transient receptor potential ankyrin 1, (+) = excitation, (−) = inhibition, EPSP = excitatory post-synaptic potential. LID = lidocaine, QUR = Quercetin.

In summary, our investigation demonstrated that local EGCG administration attenuates hyperexcitability of nociceptive trigeminal ganglion primary sensory neurons innervating inflamed tissues. This provides a potential therapeutic avenue for the management of trigeminal inflammatory pain, likely with minimal adverse effects, thereby advancing the understanding and application within the domain of CAM. The compelling evidence from this study strongly suggests that EGCG, a phytochemical, functions as a more potent local analgesic compared to Nav channel blockers, evidenced by its inhibition of both generator potentials and action potentials in nociceptive primary nerve terminals (Figure 7). However, further comprehensive studies are warranted to precisely elucidate the molecular targets mediating EGCG’s analgesic effects.

## 5. Conclusions

Our findings clearly demonstrate that locally injected EGCG suppresses hyperexcitability in nociceptive primary sensory neurons of the inflamed TG. This phytochemical, when administered locally, acts as a more potent local analgesic than voltage-gated sodium channel blockers. Its efficacy stems from its ability to inhibit both generator potentials—likely through ASIC blockade—and action potentials in nociceptive primary nerve terminals. This research significantly advances the field of CAM. Continued research is vital to fully elucidate EGCG’s mechanism of action and ensure its safety profile.

## Data Availability

All data from this study are included in the main body of the article.

## Abbreviations

CAM	Complementary alternative medicine
SpVc	Spinal trigeminal nucleus caudalis
TG	Trigeminal ganglion
DRG	Dorsal root ganglion
WDR	Wide dynamic range
EGCG	(-)-epigallocatechin-3-gallate
ASICs	Acid sensing ionic channels
Nav	Voltage-gated Na
Kv	Voltage-gated K
Cav	Voltage-gated Ca
TTX-S	Tetrodotoxin-sensitive
TTX-R	Tetrodotoxin-resistant
CFA	Freund’s adjuvant
K-current	Slow-inactivating sustained K
A-current	Fast-activating transient
TMJ	Temporomandibular joint
